# Survival of Patients With Cervical Cancer Treated With Definitive Radiotherapy or Concurrent Chemoradiotherapy According to Histological Subtype: A Systematic Review and Meta-Analysis

**DOI:** 10.3389/fmed.2022.843262

**Published:** 2022-03-01

**Authors:** Guorong Yao, Jian Qiu, Fengjia Zhu, Xiaoxie Wang

**Affiliations:** Department of Gynaecology and Obstetrics, Huzhou Central Hospital, Affiliated Central Hospital Huzhou University, Huzhou, China

**Keywords:** cervical cancer survival cervical cancer, chemotherapy, radiotherapy, disease-free survival, overall survival

## Abstract

**Background:**

Cervical cancer is a leading cause of morbidity and mortality for women worldwide. Different histopathological cervical cancer subtypes (i.e., adenocarcinoma/adenosquamous carcinoma, and squamous cell carcinoma) are all treated similarly with definitive radiotherapy or concurrent chemoradiotherapy, but studies have reported differing survival prognoses. In this review and meta-analysis, we compared the disease-free and overall survivals of patients with cervical cancer treated with definitive radiotherapy or concurrent chemoradiotherapy according to the histopathological subtypes.

**Objective:**

To compare the disease-free and overall survivals of patients with adenocarcinoma/adenosquamous carcinoma and squamous cell carcinoma cervical cancer treated with definitive radiotherapy or concurrent chemoradiotherapy.

**Methods:**

We systematically searched the Web of Science, EMBASE, CENTRAL, Scopus, and MEDLINE academic databases following PRISMA guidelines. We identified publications to conduct a random-effects meta-analysis to evaluate the disease-free and overall survivals of patients with cervical adenocarcinoma/adenosquamous carcinoma and squamous cell carcinoma treated with definitive radiotherapy or concurrent chemoradiotherapy.

**Results:**

From 963 studies, we found eight eligible ones with 13,859 patients with cervical cancer (mean age, 52.2 ± 7.9 years). Our meta-analysis revealed a poorer outcome of disease-free (hazard ratio, 1.51; 95% CI, 1.28–1.79) and overall (hazard ratio 1.41; 95% CI, 1.26–1.57) survivals for patients with adenocarcinoma/adenosquamous carcinoma undergoing definitive radiotherapy or concurrent chemoradiotherapy than for those with squamous cell carcinoma undergoing similar treatments. We also observed that larger tumor size and advanced tumor stage are also significant prognostic factors that adversely impact survival outcomes in cervical cancer patients undergoing definitive radiotherapy or concurrent chemoradiotherapy.

**Conclusion:**

Our results show poor disease-free and overall survivals for patients with cervical cancer and adenocarcinoma/adenosquamous carcinoma than for those with squamous cell carcinoma after treatment with definitive radiotherapy or concurrent chemoradiotherapy. Our findings clarify the risks associated with the conventional management of cervical cancer according to the histological type.

## Introduction

Cervical cancer is the fourth most common type of cancer in women (1, 2). According to the American Cancer Society, the malignancy originates in the cellular lining of the cervix, and most cases can be classified as being squamous cell carcinomas, with adenocarcinoma/adenosquamous carcinomas following the list (3, 4). Epidemiological studies have reported a high incidence of cervical cancer (almost 40%) (5, 6), and the World Health Organization acknowledges that almost 310,000 women worldwide perish annually due to cervical cancer (2).

Past decades have largely seen a reduction in the incidence of the commonly occurring squamous cell carcinoma-based cervical cancer (7). This decrease in incidence has been attributed to the development of advanced cytological screening procedures that allow clinicians to preemptively treat the malignancy in its precancerous stages (8–10). Despite the efficacy of such widespread screening programs, they are ineffective in detecting the other histopathological cervical cancer variants, and the overall incidence of cervical adenocarcinoma/adenosquamous carcinomas has increased worldwide (11–13). Randomized controlled trials have led to the use of concurrent chemotherapy alongside radiotherapy as the standard treatment for all the cervical cancer histopathological subtypes (14–16). However, this may not be the best approach (17, 18). However, some evidence suggests that this standard treatment may not be the best for patients with adenocarcinoma/adenosquamous carcinoma-based cervical cancer (19, 20).

Many cohort studies have compared the disease-free and overall survivals in patients with adenocarcinoma/adenosquamous carcinoma and squamous cell carcinoma (17, 18, 20–24), their results differ as to the impact of the histopathological subtypes on the overall survival in these patients after definitive radiotherapy or concurrent chemoradiotherapy. Some studies found worse survivals for patients with adenocarcinoma/adenosquamous carcinoma than for those with squamous cell carcinoma (18, 20, 21). But others found the worse survivals in the patients with squamous cell carcinoma (17, 22–24). Similarly, whether the disease-free survival differs among the patients with cervical cancer based on the histological type also remains unclear. Some studies found a stronger negative impact on the disease-free survival of patients with adenocarcinoma/adenosquamous carcinoma than on those with squamous cell carcinoma (18, 20, 21), but others reported a lack of statistically significant differences (17, 23, 24).

To the best of our knowledge, no systematic review or meta-analysis before this one has compared the survivals after definitive radiotherapy or concurrent chemoradiotherapy in patients with cervical cancer and either adenocarcinoma/adenosquamous carcinoma or squamous cell carcinoma. Therefore, we will attempt to synthesize the evidence on the disease-free and overall survivals of patients with cervical cancer according to their histopathological subtypes. Our findings should clarify the survival prognoses of the different cervical cancer subtypes after definitive radiotherapy or concurrent chemoradiotherapy.

## Methods

We adhered to the PRISMA (Preferred Reporting Items for Systematic Reviews and Meta-Analyses) guidelines (25) to conduct this meta-analysis.

### Data Search Strategy

We searched the literature in five scientific databases (Web of Science, MEDLINE, CENTRAL, EMBASE, and Scopus) from inception until April 2021. The search was performed using a combination of MeSH keywords including “adenocarcinoma,” “adenosquamous carcinoma,” “squamous cell carcinoma,” “cervical cancer,” “radiation therapy,” “concurrent chemoradiotherapy,” included “disease-free survival,” and “overall survival.” We also manually searched the bibliography section of the studies to identify all relevant studies. The inclusion criteria follow:

a) Studies comparing the disease-free and/or overall survivals in between cervical cancer patients with adenocarcinoma/adenosquamous carcinoma and squamous cell carcinoma.
b) Studies with cervical cancer patients receiving radiation therapy and concurrent chemoradiotherapy.
c) Studies conducted in human participants.
d) Studies published in peer-reviewed scientific journals.
e) Studies published in English language.

Case series, case reports, conference proceedings and abstracts, letters to the editor, opinion papers, theses, reviews, and meta-analyses were not considered for this review. The screening of the studies was independently performed by two reviewers. Disagreements were resolved by discussion with a third independent reviewer.

### Quality Assessment

We assessed the risk of bias in the included studies using the Newcastle Ottawa scale (26). This tool evaluates the outcomes for selective reporting, confounding bias, measurement of outcomes, and incomplete data availability as bias threats. In addition, two reviewers independently appraised the methodological quality of the studies. Here, again disagreements were resolved after the intervention of a third reviewer to arbitrate.

### Data Analysis

We used the Comprehensive Meta-analysis version 2.0 (CMA) software (27) to perform a within-group random-effects model meta-analysis (28). We calculated the hazard ratios to assess disease-free and overall survivals of patients with adenocarcinoma/adenosquamous carcinoma or squamous cell carcinoma. We assessed the heterogeneity among the studies by computing *I*^2^ statistics; we considered values between 0 and 25% as indicative of negligible heterogeneity, values between 25 and 75% as moderately heterogeneous, and values ≥75% as substantially heterogeneous (29). We converted medians and ranges in individual studies into means and standard deviations using the method of Hozo et al. (30). Moreover, we applied Duval and Tweedy's trim and fill procedure (31) to evaluate publication bias. This publication bias analysis is characterized by the imputation of studies from either side of a plotted graph to identify any unbiased effects. The significance level for this study was determined at 5%.

## Results

**Figure 1:**
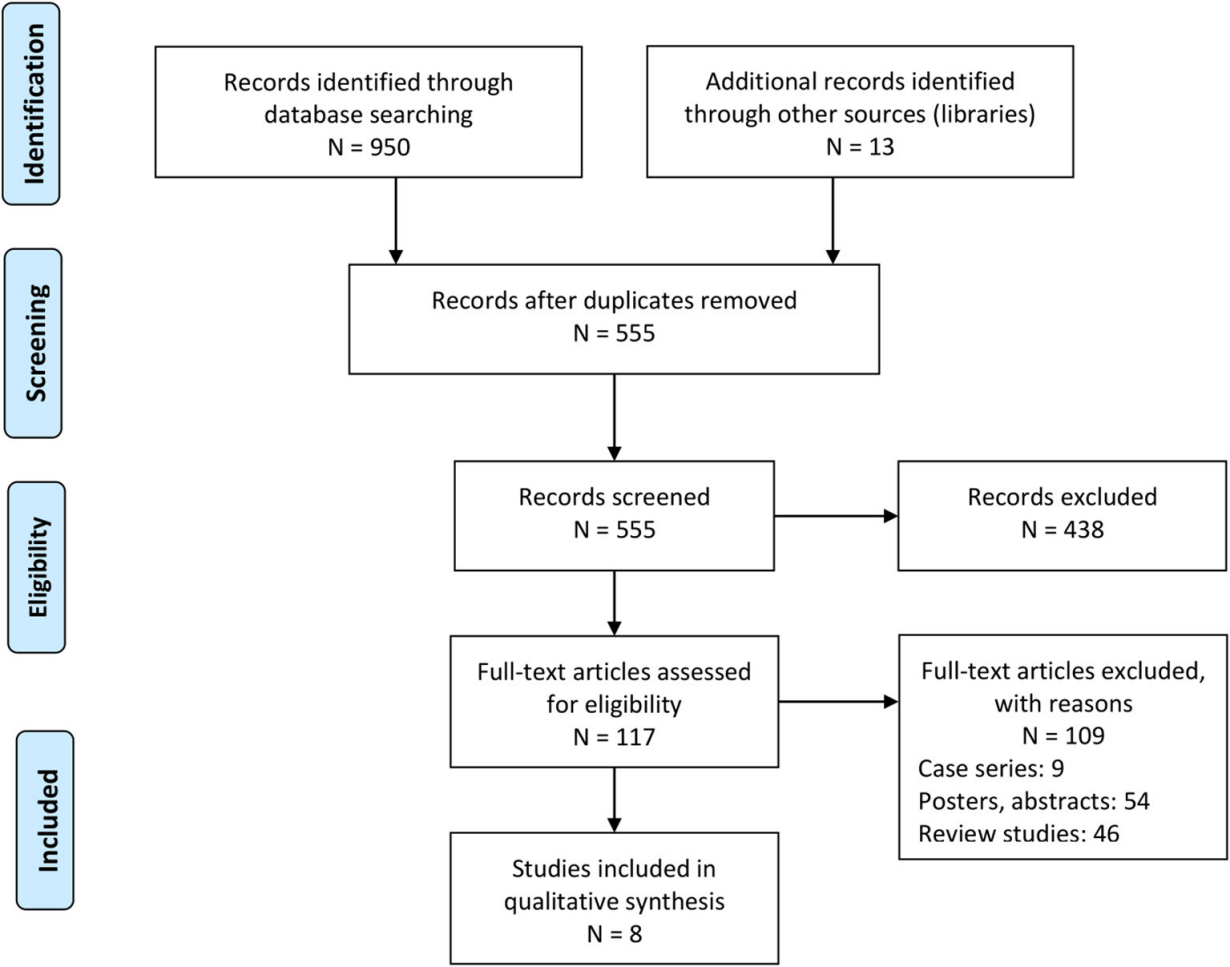
PRISMA flowchart.

Our literature search provided a total of 950 studies. We identified an additional 13 during the screening of the reference sections of the included studies (Figure 1). After applying our inclusion criteria, we obtained eight retrospective cohort studies (17, 18, 20–24, 32). We extracted the data into tables (see summary in Table 1).

**Table 1:** Details of the studies included in the meta-analysis.

**Study**	**Country**	**Type of study**	**Sample descriptive**	**Age (M ±SD years)**	**Tumor stage**	**Tumor size (cm)**	**Duration of radiation therapy (days)**	**Follow-up (months)**	**Disease-free survival % (95% CI)**	**Overall survival % (95% CI)**
Kang et al. (21)	South Korea	Retrospective cohort study	SCC: 354 AC: 44	SCC: 57.4 ± 12.0 AC: 56.5 ± 12.2	SCC II:206 III:110 IV:38 AC II:26 III:13 IV:5	SCC ≥4:84 <4:270 AC ≥4:13 <4:31	SCC: 54 AC: 53	60	–	–
Hu et al. (18)	China	Retrospective cohort study	SCC: 744 AC: 71	–	SCC I:92 II:505 III-IV:147 AC I:7 II:54 III-IV:10	SCC ≥4:457 <4:287 AC ≥4:42 <4:29	–	36.2	SCC: 77.5% AC: 57.3%	SCC: 85.2% AC: 75.4%
Zhou et al. (24)	China	Retrospective cohort study	SCC: 7,530 AC: 925 ASC: 296	52 (19–98)	SCC I:1,589 II:3,487 III:2,235 IV:219 AC I:296 II:414 III:190 IV:25 ASC I: 79 II:141 III:64 IV:12	–	–	39	SCC: 59.3% AC/ASC: 53.9%	SCC: 51.1% AC/ASC: 40.3%
Yokoi et al. (20)	Japan	Retrospective cohort study	SCC: 225 AC/ASC: 24	SCC: 61.4 ± 12.9 AC/ACS: 62.6 ± 12.4	SCC II:81 III:129 IV:15 AC/ACS II:15 III:7 IV:2	SCC >4:167 ≤ 4:58 AC/ACS >4:17 ≤ 4:7	SCC: 45 AC/ACS: 47.5	60	–	SCC: 58.6% AC/ACS: 26.7%
Rose et al. (23)	Canada	Retrospective cohort study	SCC: 1,489 AC/ASC: 182	SCC: 46.5 AC/ASC: 46	SCC II:1,000 III:446 IV:43 AC/ACS II:136 III:42 IV:4	SCC ≥5:1,243 <5:234 AC/ACS ≥5:41 <5:141	–	120	SCC: 52% AC/ASC: 53.2%	SCC: 48.2% AC/ASC: 50.5%
Chen et al. (17)	Taiwan	Retrospective cohort study	SCC: 194 AC/ASC: 35	SCC: 63 AC/ASC: 35	SCC II:134 III:50 IV:10 AC/ACS II:26 III:6 IV:3	SCC ≥5:92 <5:102 AC/ACS ≥5:11 <5:24	–	60	SCC: 47.6% AC/ASC: 30%	SCC: 58.1% AC/ASC: 41.3%
Noh et al. (32)	South Korea	Retrospective cohort study	SCC: 1,073 AC: 185 ASC: 65	SCC: 51 AC: 46 ASC: 50	SCC I:757 II:316 AC I:148 II:37 ASC I:58 II:7	–	–	60	–	SCC: 87.6% AC: 75.8% ASC: 83.2%
Katanyoo et al. (22)	Thailand	Retrospective cohort study	SCC: 282 ASC: 141	SCC: 50.8 ± 10.7 ASC: 49.1 ± 10.3	SCC II:170 III:110 IV:2 AC II:85 III:55 IV:1	SCC >4:130 ≤ 4:152 AC >4:68 ≤ 4:73	–	60	–	SCC: 59.9% ASC: 61.1%

*M, mean; SD, standard deviation; F, woman; M, man; SCC, squamous cell carcinoma; AC/ASC, adenocarcinoma/adenosquamous carcinoma*.

### Participant Information

We included data from 13,859 women in the eight studies included. We found 11,891 patients with squamous cell carcinoma and 1,968 with adenocarcinoma/adenosquamous carcinoma.

The average age of the participants was as 52.2 ± 7.9 years. The average age of patients with squamous cell carcinoma was 55.01 ± 6.58 years, and the average age of patients with adenocarcinoma/adenosquamous carcinoma was 49.8 ± 8.6 years. Two studies failed to report the ages of their patients (18, 24).

### Quality Assessment for Cohort Studies

**Figure 2:**
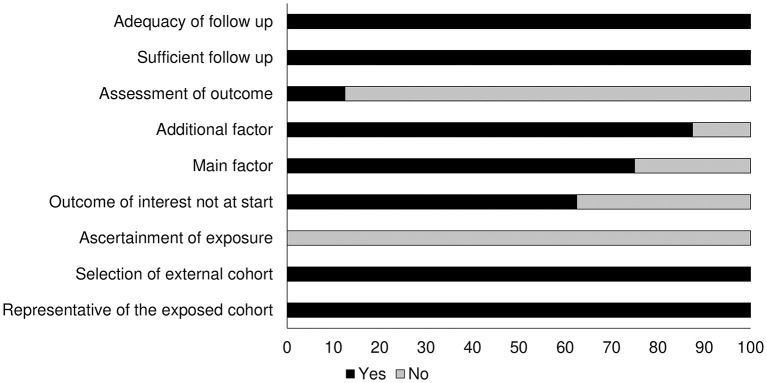
Risk of bias according to the Newcastle Ottawa scale for cohort studies.

Table 2 shows the results of the risk of bias obtained with the Newcastle Ottawa scale. We found the overall risk to be low (see also the graph on Figure 2).

**Table 2:** Risk of bias for individual studies based on the Newcastle Ottawa scale.

**Study**	**Selection**			**Comparability**			**Outcome**			**Total**
	**Exposed cohort representative**	**External cohort selection**	**Exposureascertainment**	**Outcome of interestabsent at start**	**Main factor**	**Additional factors**	**Outcome assessment**	**Sufficient follow-up**	**Follow-up adequacy**	**(9/9)**
Kang et al. (21)	+	+	0	+	+	+	0	+	+	7
Hu et al. (18)	+	+	0	0	+	+	0	+	+	6
Zhou et al. (24)	+	+	0	+	+	0	+	+	+	7
Yokoi et al. (20)	+	+	0	+	+	+	0	+	+	7
Rose et al. (23)	+	+	0	+	+	+	0	+	+	7
Chen et al. (17)	+	+	0	+	+	+	0	+	+	7
Noh et al. (32)	+	+	0	0	+	+	0	+	+	6
Katanyoo et al. (22)	+	+	0	0	0	+	0	+	+	5

### Publication Bias

**Figure 3:**
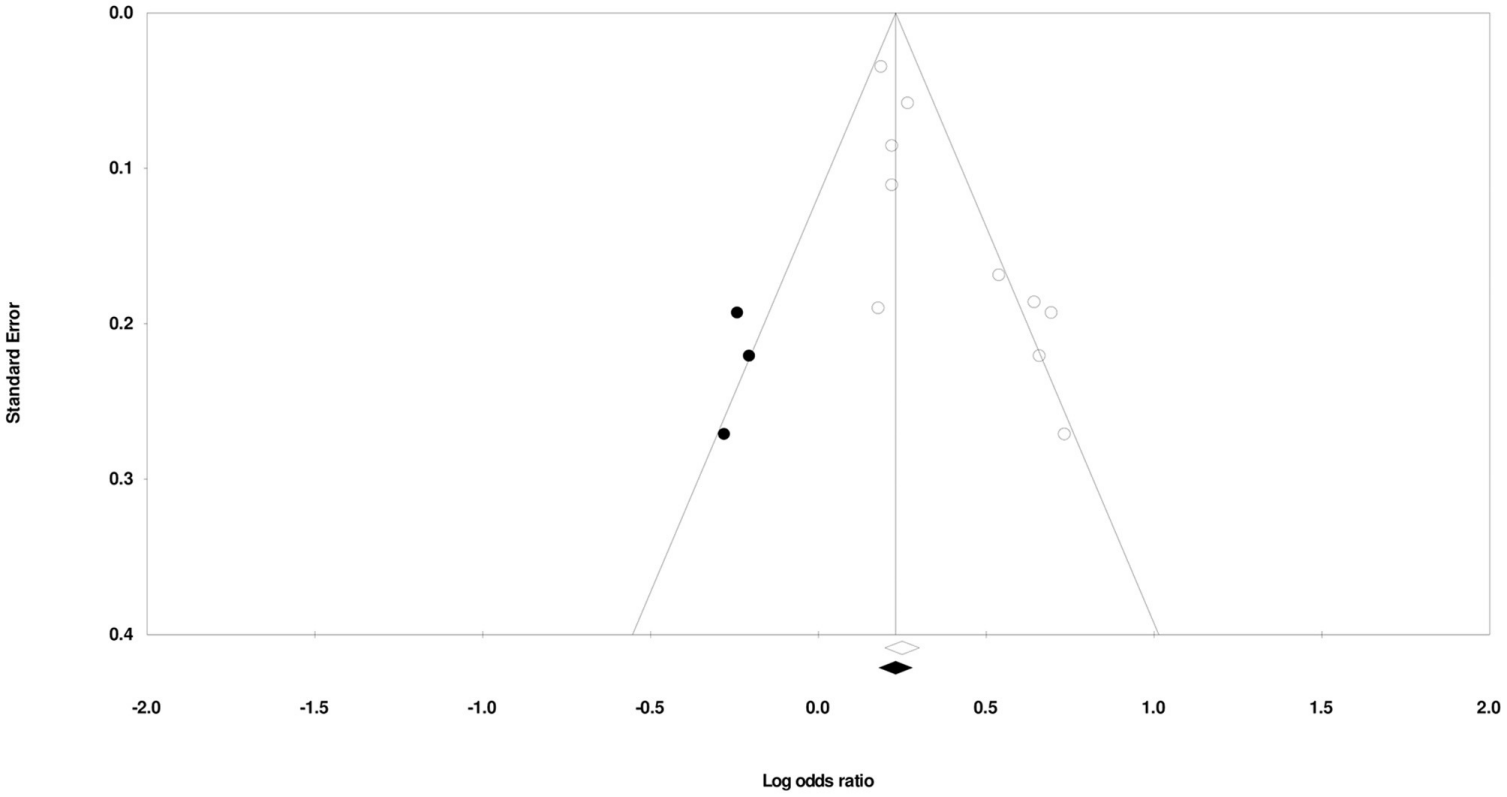
Publication bias by Duval and Tweedy's trim and fill method.

We used Duval and Tweedy's trim and fill method to determine whether studies were missing on either side of the mean effect of the funnel plot due to publication bias. We identified three studies missing on the left side of the mean effect. The overall random effect models determined the point estimate (1.41) and the 95% CI (1.26–1.57) for all the combined studies; after using the trim and fill method, the imputed point estimate was 1.32 and the 95% CI (1.18–1.48). Figure 3 shows the publication bias graph.

### Meta-Analysis Report

#### Disease-Free Survival

**Figure 4:**
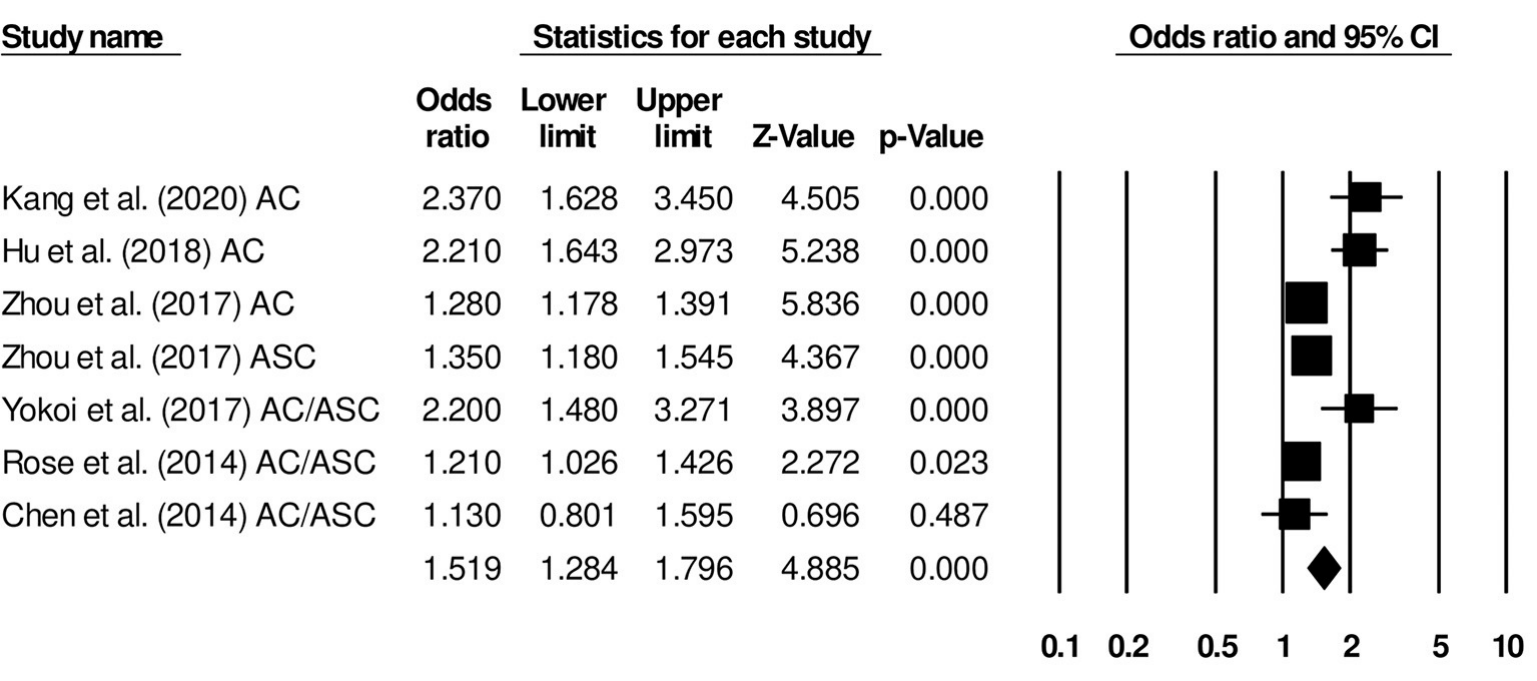
Forest plot for studies evaluating the disease-free survival in patients with adenocarcinoma/adenosquamous carcinoma or squamous cell carcinoma.

Six studies in our analysis reported disease-free survival statistics. We observed an increased hazard ratio in patients with adenocarcinoma/adenosquamous carcinoma than in patients with squamous cell carcinoma (Figure 4; hazard ratio, 1.51; 95% CI, 1.28–1.79; *p* = 0.001) with moderate heterogeneity (*I*^2^: 44.2%).

**Figure 5:**
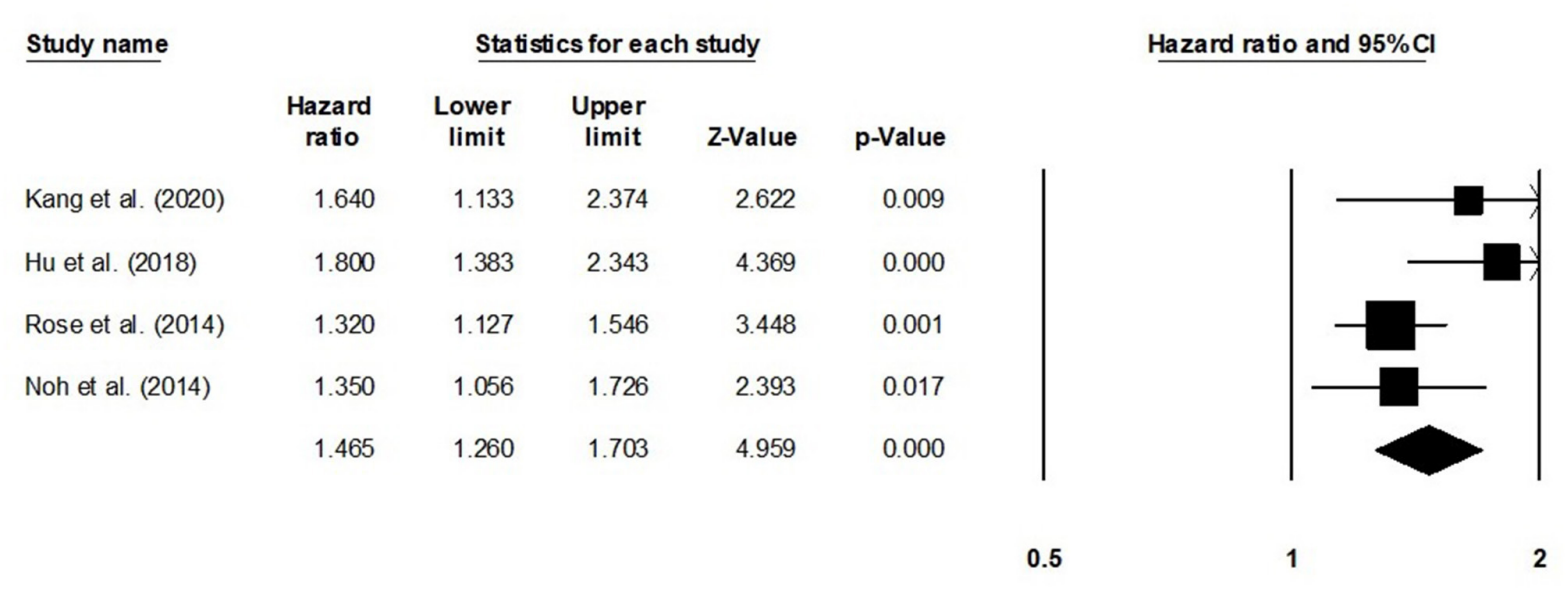
Forest plot for studies evaluating the disease-free survival in patients with differential tumor size.

**Figure 6:**
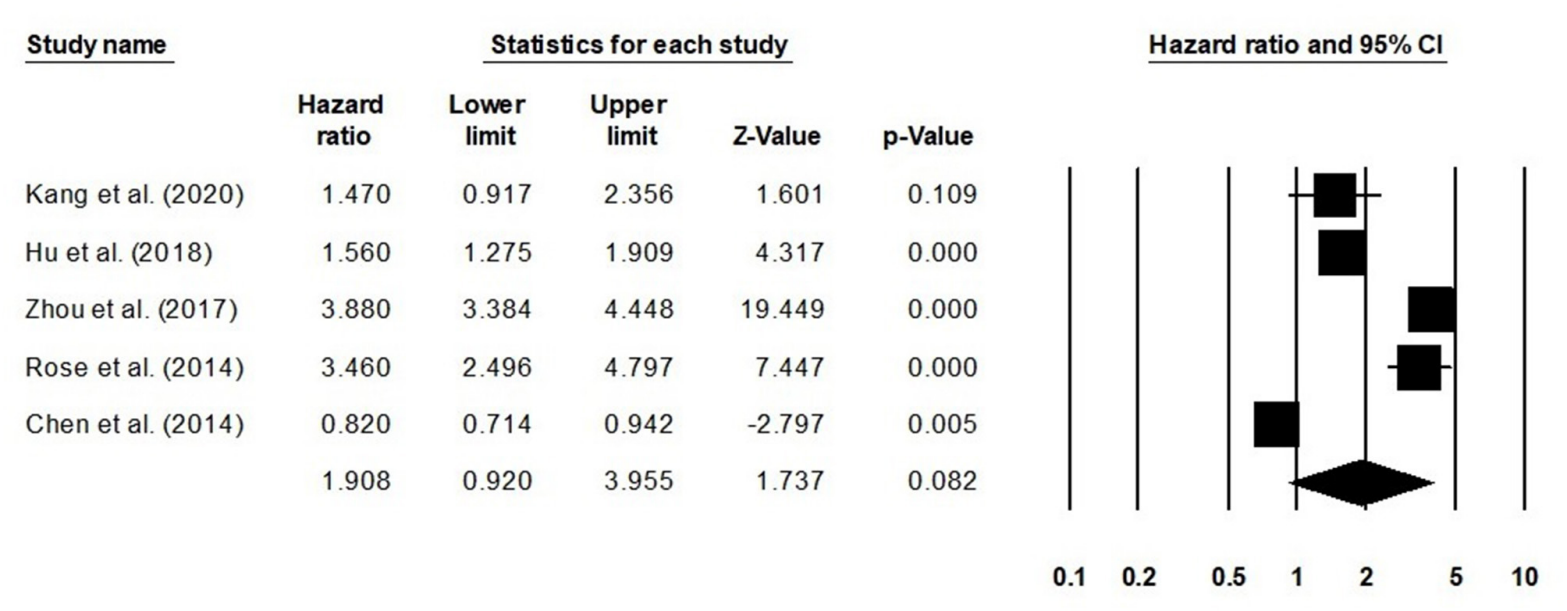
Forest plot for studies evaluating the disease-free survival in patients with differential tumor stage.

We also conducted further subgroup analysis in which we evaluated disease-free survival outcome based on tumor size and the stage of tumor. Four studies had reported disease-free survival outcome based on the tumor size. We observed an increase hazard ratio in patients with larger tumor size than in patients with smaller (Figure 5; hazard ratio, 1.46; 95% CI, 1.26–1.70; *p* = 0.001) with no heterogeneity (*I*^2^: 0%). Five studies had reported disease-free survival outcome based on the tumor stage. We observed an increase hazard ratio in patients with higher tumor stage than in patients with lesser stage (Figure 6; hazard ratio, 1.90; 95% CI, 0.92–3.95; *p* = 0.08) with no heterogeneity (*I*^2^: 0%).

#### Overall Survival

**Figure 7:**
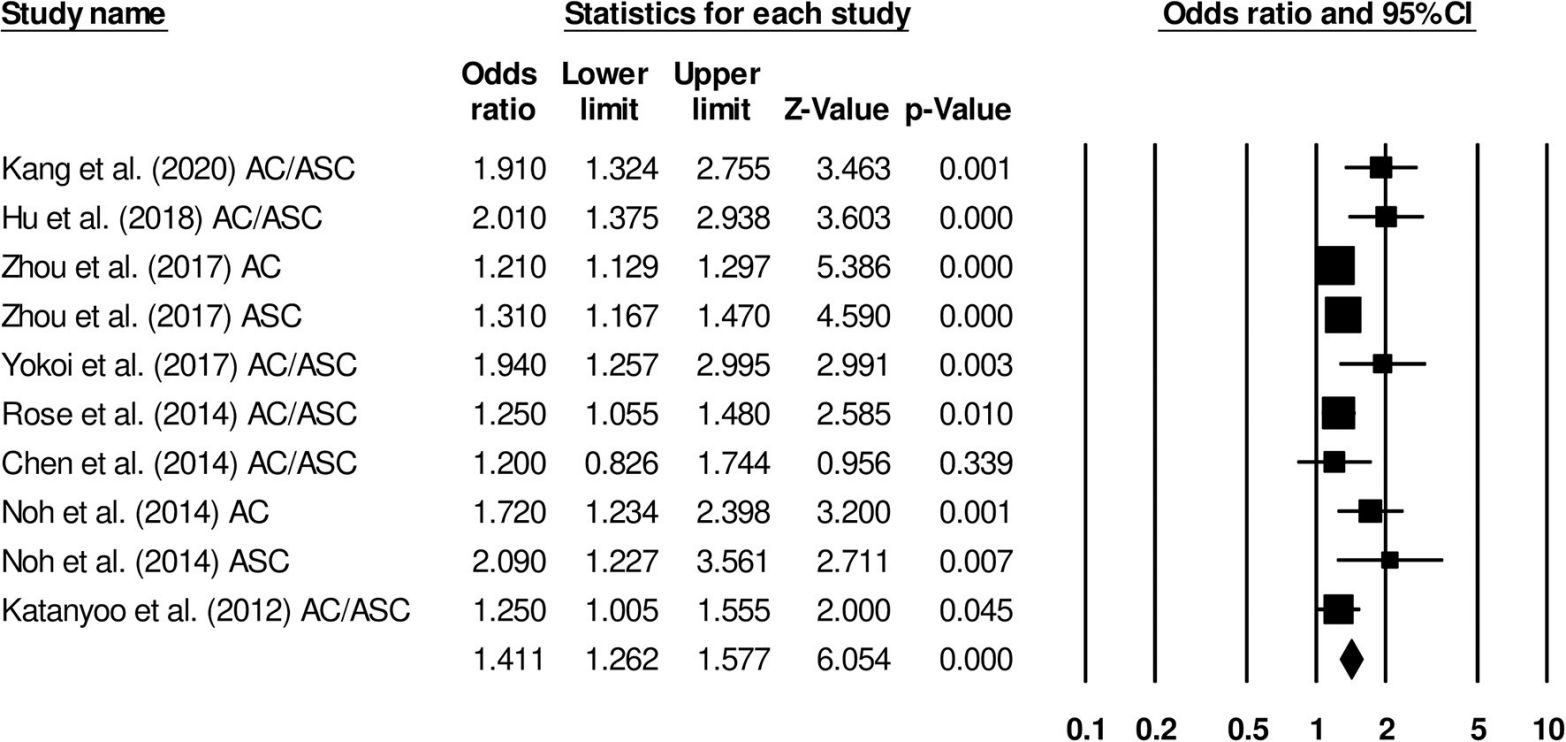
Forest plot for studies evaluating the overall survival in patients with adenocarcinoma/adenosquamous carcinoma or squamous cell carcinoma.

The overall survival was reported by eight studies. We observed an increased hazard ratio in patients with adenocarcinoma/adenosquamous carcinoma than in patients with squamous cell carcinoma (Figure 7) (hazard ratio, 1.41; 95% CI, 1.26–1.57; *p* < 0.001) with moderate heterogeneity (*I*^2^: 24.9%).

**Figure 8:**
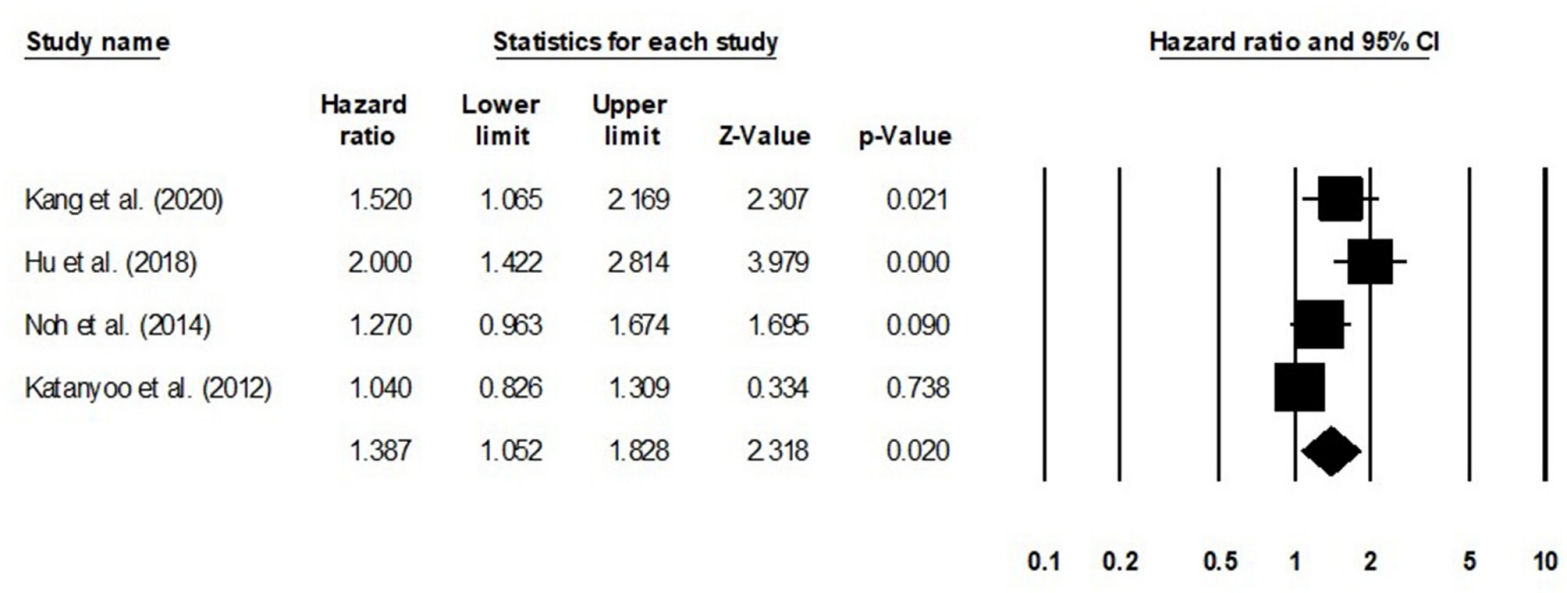
Forest plot for studies evaluating the overall survival in patients with differential tumor size.

**Figure 9:**
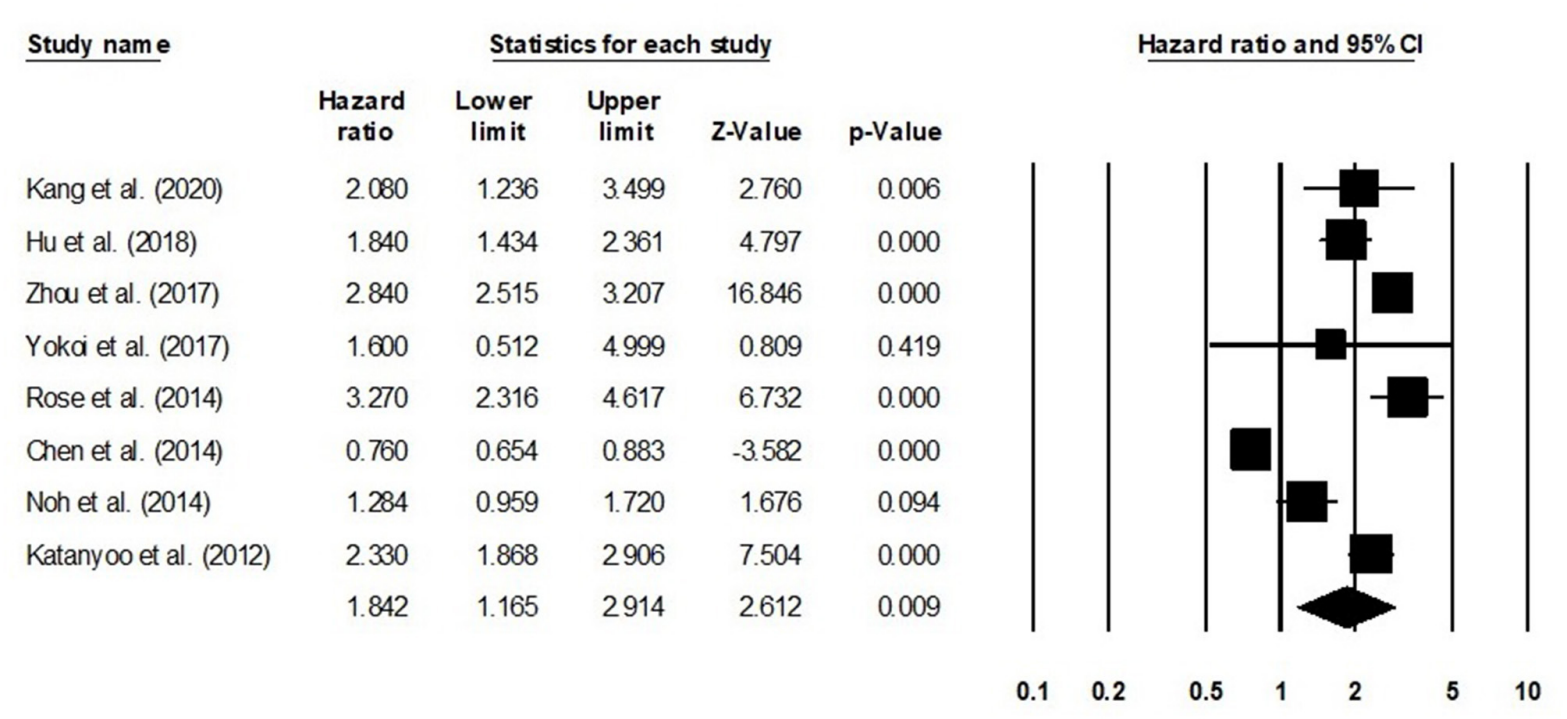
Forest plot for studies evaluating the overall survival in patients with differential tumor stage.

We conducted further subgroup analysis in which we evaluated overall survival outcome based on tumor size and the stage of tumor. Four studies had reported overall survival outcome based on the tumor size. We observed an increase hazard ratio in patients with larger tumor size than in patients with smaller (Figure 8; hazard ratio, 1. 38; 95% CI, 1.05–1.82; *p* = 0.02) with no heterogeneity (*I*^2^: 0%). Eight studies had reported overall survival outcome based on the tumor stage. We observed an increase hazard ratio in patients with higher tumor stage than in patients with lesser stage (Figure 9; hazard ratio, 1.84; 95% CI, 1.16–2.91; *p* = 0.009) with no heterogeneity (*I*^2^: 0%).

## Discussion

We present evidence pointing to a difference in the survival of patients with cervical cancer undergoing definitive radiotherapy or concurrent chemoradiotherapy based on their histological type. We found poorer disease-free and overall survivals for patients with adenocarcinoma/adenosquamous carcinoma than for those with squamous cell carcinoma after definitive radiotherapy or concurrent chemoradiotherapy. We also observed that larger tumor size and advanced tumor stage are also significant prognostic factors that adversely impacts survival outcomes in in cervical cancer patients undergoing definitive radiotherapy or concurrent chemoradiotherapy.

Cervical cancer management is challenging because of its poor prognosis and various manifestations (33–35). Patients with cervical cancer are often treated with a similar approach (i.e., concurrent chemo- and radiotherapy) (14–16) regardless of the cancer's histopathological subtype. Some studies have suggested that patients with adenocarcinoma/adenosquamous cervical carcinoma do not fare as well as those with the squamous cell carcinoma subtype when treated with the standard therapy (18, 22). Moreover, some evidence suggests that the patients with adenocarcinoma/adenosquamous cervical carcinoma treated with the standard therapy may exhibit higher morbidity (high failure and relapse rates, lymph node metastases) and mortality rates (18) than their counterparts with squamous cell cervical carcinoma after the same treatments.

We observed that all the studies included had reported poorer disease-free survivals for the patients with adenocarcinoma/adenosquamous carcinoma than for those with squamous cell carcinoma-based cervical cancer. In a cohort representative of the Chinese population, Zhou et al. (24) found poorer disease-free survival in patients with adenocarcinoma/adenosquamous cervical carcinoma than in those with squamous cell cervical carcinoma. The authors attributed their findings and those by Huang et al. (36) to the radioresistant properties of adenocarcinoma/adenosquamous carcinomas, which prevent the cancer's complete cure, and ultimately affect the survival of the patients. Similarly, Hu et al. (18) also found a poorer 3-year disease-free survival rate for the adenocarcinoma group (53.7%) than that for the squamous cell carcinoma group (77.5%), and they confirmed their findings with a propensity score match among 142 patients. Importantly, these authors reported a trend toward improved survival for the adenocarcinoma group treated with paclitaxel, and they recommended a focus on this trend for future studies with large sample sizes. We confirmed these findings and report poorer disease-free survival in patients with adenocarcinoma/adenosquamous carcinoma than that in patients with squamous cell carcinoma-based cervical cancer (HR, 1.51) after definitive radiotherapy or concurrent chemoradiotherapy.

In terms of the overall survivals after the standard treatment for these cervical cancer types, we found a lack of consensus in the included studies. In a retrospective cohort study, Rose et al. (23) reported similar overall survivals among 1,671 patients with advanced-stage cervical cancer (*p* = 0.45) regardless of the cancer subtype group and specially for the patients receiving cisplatin-based chemoradiation. Similarly, Katanyoo et al. (22) also reported non-significant differences (0.13) in the 5-year overall survivals between adenocarcinoma and squamous cell carcinoma groups receiving radiation therapy and concurrent chemotherapy. The authors mentioned that the similar overall survivals between the two histological subtypes remained even when they compared the tumor stages and sizes, and their respective treatments. On the other hand, Yokoi et al. (20) and Hu et al. (18) reported improved overall survivals for the squamous cell carcinoma group than for the adenocarcinoma/adenosquamous carcinoma group. They also mentioned that the patients with adenocarcinoma/adenosquamous cervical carcinoma had higher failure rates and relapse risks after definitive radiotherapy or concurrent chemoradiotherapy (18). Our results support those findings showing poorer overall survivals in patients with adenocarcinoma/adenosquamous cervical carcinoma than in those with squamous cell cervical carcinoma (HR, 1.41) after definitive radiotherapy or concurrent chemoradiotherapy.

Our study had some limitations. First, we were not able to pre-register this study in a systematic review repository such as PROSPERO York or Joanna Briggs Institute. We understand that this may raise concerns on the validity of our findings (37). However, we assure our readers that we made several attempts to register this review, but failed due to the extended registration times at the repositories owing to the COVID-19 pandemic. Second, all the included studies in our review were retrospective cohort studies, as a result we cannot exclude potential sources of biases. Third, our study did not evaluate the potential role of surgery in the evaluated outcomes. Fourth, the included studies reported survivals after differing follow-up periods after the definitive radiotherapy or concurrent chemoradiotherapy: Five studies reported the comparative survival outcomes after 5 years (17, 20–22, 32), two after 3 years (18, 24), and one after 10 years (23). Therefore, we cannot rule out bias in our findings. Future studies are needed to address these limitations and validate our results.

In conclusion, we found survival differences among patients with cervical cancer after definitive radiotherapy or concurrent chemoradiotherapy based on the cancer's histological subtype, tumor size, and tumor stage. These findings should be useful for clinicians because they synthesize the available evidence on the prognoses of the different cervical cancer subtypes after the standard management.

## Data Availability Statement

The original contributions presented in the study are included in the article/supplementary material, further inquiries can be directed to the corresponding author/s.

## Author Contributions

GY and JQ: conceptualized and designed the study. GY and FZ: did literature search and data collection. JQ and XW: analyzed the data. GY: wrote the paper. FZ and XW: reviewed and edited the manuscript. All authors read and approved the final manuscript.

## Funding

The current study was funded by Huzhou Science and Technology Bureau (Grant Number: 2018GY01).

## Conflict of Interest

The authors declare that the research was conducted in the absence of any commercial or financial relationships that could be construed as a potential conflict of interest.

## Publisher's Note

All claims expressed in this article are solely those of the authors and do not necessarily represent those of their affiliated organizations, or those of the publisher, the editors and the reviewers. Any product that may be evaluated in this article, or claim that may be made by its manufacturer, is not guaranteed or endorsed by the publisher.
